# Cellular Senescence: Molecular Targets, Biomarkers, and Senolytic Drugs

**DOI:** 10.3390/ijms23084168

**Published:** 2022-04-10

**Authors:** Natalie Kudlova, Juan Bautista De Sanctis, Marian Hajduch

**Affiliations:** 1 Institute of Molecular and Translational Medicine, Faculty of Medicine and Dentistry, Palacky University, 77147 Olomouc, Czech Republic; natalie.taborska@upol.cz (N.K.); juan.desanctis@upol.cz (J.B.D.S.); 2 Institute of Molecular and Translational Medicine Czech Advanced Technologies and Research Institute, Palacky University, 77147 Olomouc, Czech Republic

**Keywords:** senescence, aging, cellular model, mouse model, senolytics

## Abstract

Cellular senescence is defined as irreversible cell cycle arrest caused by various processes that render viable cells non-functional, hampering normal tissue homeostasis. It has many endogenous and exogenous inducers, and is closely connected with age, age-related pathologies, DNA damage, degenerative disorders, tumor suppression and activation, wound healing, and tissue repair. However, the literature is replete with contradictory findings concerning its triggering mechanisms, specific biomarkers, and detection protocols. This may be partly due to the wide range of cellular and in vivo animal or human models of accelerated aging that have been used to study senescence and test senolytic drugs. This review summarizes recent findings concerning senescence, presents some widely used cellular and animal senescence models, and briefly describes the best-known senolytic agents.

## 1. Introduction

Senescence and aging are closely connected but not synonymous processes. Aging is a predictable natural gradual process of the organism and occurs due to pro-aging mechanisms such as DNA damage, peroxidation, protein misfolding, resulting in cell death or senescence. Senescence is the process of the stable and irreversible growth arrest of cells. This process contributes to aging and age-related diseases, but also physiologically protects multicellular organisms from neoplasia [1]. 

**Figure 1:**
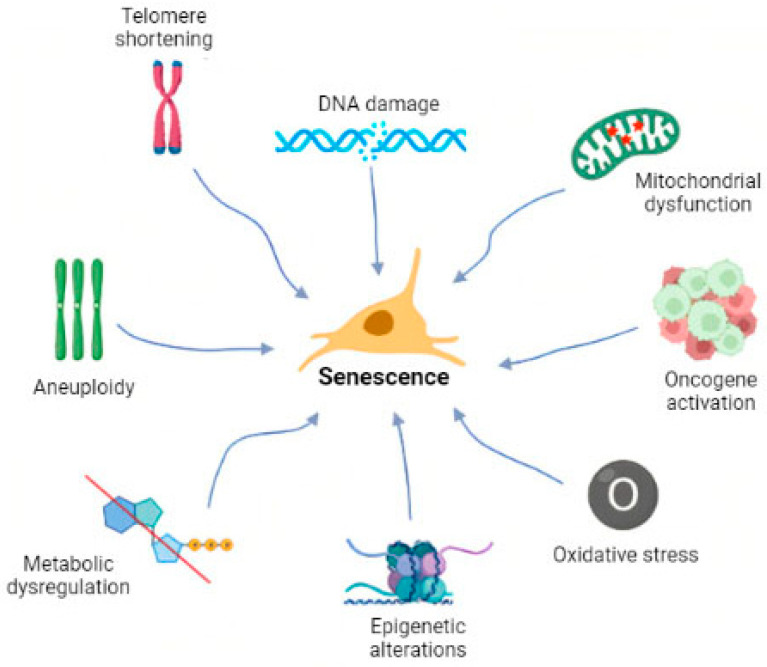
Cellular senescence can be induced by several stimuli. The first to be identified was telomere shortening, which is closely connected to so-called replicative senescence. Genomic changes, including DNA damage, oncogene activation, aneuploidy, and epigenetic changes, can also lead to senescence. Other factors that may contribute to irreversible cell cycle arrest include mitochondrial dysfunction, large-scale ROS generation, and metabolic dysregulation.

The permanent arrest of proliferation at the cellular level was first studied by Hayflick and Moorehead in 1961, who showed that the lifespan of primary human cells was limited to approximately 60 divisions [2]. Since then, senescence has been a popular topic in biomedical research. Senescent cells have been described in detail along with the molecular mechanisms underlying senescence as well as associated pathways and/or possible targets [3,4,5]. More recently, there have been studies on the selective elimination of senescent cells and the subsequent tissue rejuvenation or alleviation of disease symptoms [6,7,8]. As a result of these efforts, the definition of senescence has evolved from that initially proposed by Hayflick [2] and others [9,10]. It is currently defined as a cellular condition responding to intrinsic or extrinsic factors (Figure 1), including oncogene activation, oxidative stress, mitochondrial dysfunction, irradiation, and exposure to chemotherapeutics [5]. Recently, the oxidative stress theory of aging has been broadly discussed. It is based on the hypothesis that during aging cells lose their functions due to ROS-induced damage [11].

**Figure 2:**
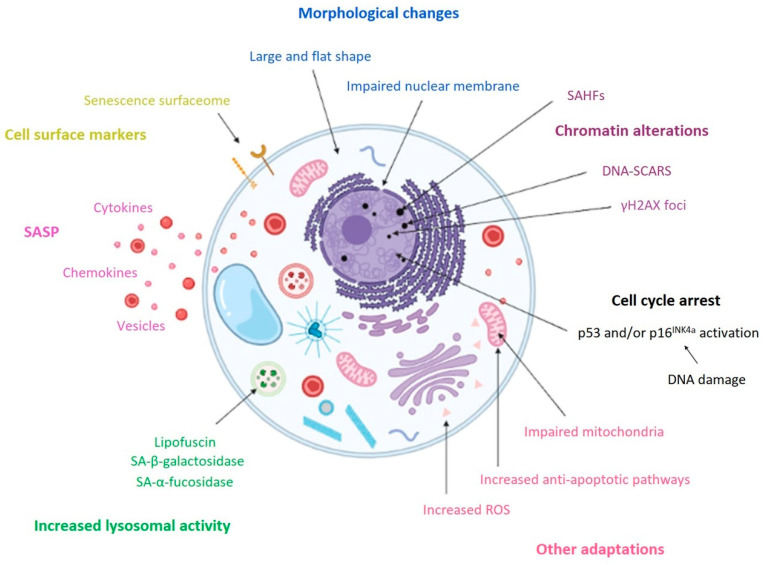
General hallmarks of cellular senescence. Markers strongly associated with senescence and seen in the majority of senescent cells include an enlarged size and flattened shape. The other features shown in this figure are not exclusive to senescence but are collectively considered hallmarks of the phenomenon. Senescent cells commonly exhibit the activation of the p53 and/or p16 pathways leading to irreversible cell cycle arrest, chromatin changes, and nuclear envelope disturbance. An increased lysosomal mass leading to enhanced SA-β-galactosidase activity is also often seen and, thus, SA-β-galactosidase activity is probably the most frequently used marker of senescence. Senescent cells may also exhibit strong paracrine secretion (SASP), severe mitochondrial disturbance associated with the activation of anti-apoptotic pathways, and other various characteristics.

The early stage of senescence is initiated in response to one or more of these stimuli. During this stage, the senescent cells undergo characteristic morphological changes, becoming flat and enlarged. This process is accompanied by nuclear chromatin remodeling and loss of lamin B1, the induction of an oxidative metabolism in mitochondria, and the activation of the senescent cells’ anti-apoptotic pathway (SCAP), making the cells resistant to death. Senescence-associated β-galactosidase activity (SA-β-gal) is also observed due to an increase in lysosomal activity, and the senescence-associated secretory phenotype (SASP) is induced. The hallmarks of senescence are illustrated in Figure 2. Autophagy is also closely connected with senescence, although its precise role is unknown [9]. Autophagy is a process of the degradation of damaged macromolecules or organelles by lysosomes in cells after stress stimuli contributing to homeostasis in organisms. It may be associated with both apoptosis and senescence [12]. Interestingly, an increased level of autophagy leads to cell death, whereas the inhibition of autophagy can trigger senescence [13,14]. For instance, autophagy in cancer cells can interfere with apoptosis [15], but in some cases it can actually cause cell death [16]. Similar contradictory evidence can be found for senescence. Autophagy is a pro-senescence inductor in some cases [17], while in other situations, impaired autophagy can also lead to senescence progression in certain cell types [18]. The majority of recent publications indicate that the outcome of autophagy and senescence interplay depends on the cell type, microenvironment, and perhaps other circumstances. The importance of senescence in organisms is controversial; it has been considered beneficial because it was suggested to be an important anti-cancer mechanism and to play key roles during embryonic development and wound healing. However, it has also been suggested that it is detrimental because it reduces the regenerative capacity of tissue, leading to aging, tissue degeneration, and cancer [10]. Both views are likely valid depending on circumstances, context, age, and health conditions.

**Figure 3:**
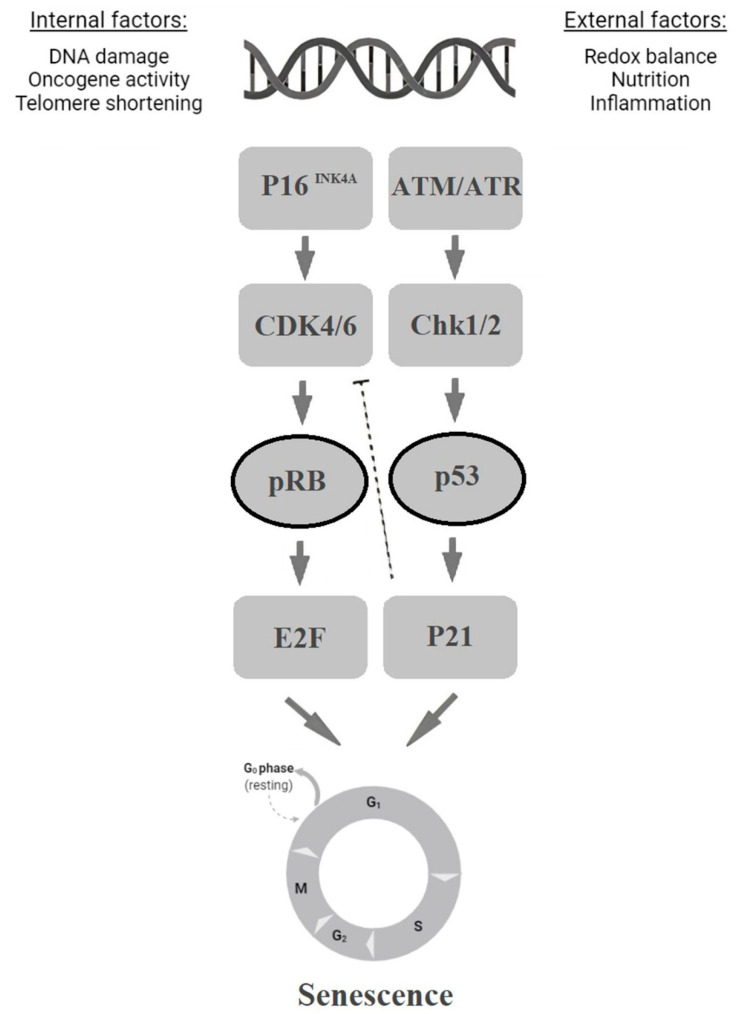
Senescence activation via the p53 and p16^INK4A^ pathways. Many stress factors can initiate senescence via the p16 and/or p53 pathways. Under specific circumstances, p16 inactivates CDK4/6, which leads to pRb accumulation and the regulation of the E2F transcription factors, resulting in senescence. DNA damage and DDR can also lead to ATM-Chk2 or ATR-Chk1 regulation with subsequent p53 and p21 activation [10].

Two major senescence-associated pathways have been highlighted in the recent literature. Both are triggered by various internal or external stimuli and the DNA damage response (DDR) and lead to the activation of the p53 and/or p16^INK4A^ pathways. The p53 and p21 proteins are essential for the initial phase of senescence, in which cells stop dividing but remain metabolically active. The role of p53 during this phase depends on its concentration and post-translational modification as well as the microenvironment. The expression of p21, which is the main p53 effector, increases dramatically during the initial phase but then decreases as senescence progresses. P21 contributes to G1/S and G2/M cell cycle arrest by specifically modulating the activity of the p53 targets CDC25B, CDC25C, and surviving [19,20,21]. At this stage, senescence is still reversible. Once the cells pass the critical point and senescence becomes irreversible, they enter the second phase-senescence maintenance. This phase is controlled by p16^INK4a^/pRb [22,23] (Figure 3). During senescence maintenance, p16 expression increases dramatically; accordingly, recent findings have shown that it plays a major role in this phase. Specifically, p16 and p21 inhibit the activity of cyclin–CDK complexes and, thereby, regulate the phosphorylation of Rb family members and E2F target gene expression [24]. Three Rb family members involved in protein–protein interactions, Rb/p105, p107, and Rbl2/p130, are associated with senescence. In particular, senescence progression in human cells depends heavily on a complex of Rbl2/p130 and E2F-4, which regulates E2F target genes. This complex is inactivated by CDKs in response to cell cycle progression stimuli. However, if the CDKs are inhibited, the Rb proteins remain active and repress cell cycle progression [25,26,27,28]. Rb family members, and especially Rbl2/p130, thus, play important roles in cell cycle arrest and senescence maintenance. However, while the entire Rb family appears to be involved in senescence, the functions of most of its members have yet to be fully elucidated [29,30].

## 2. Senescence Biomarkers and Detection Methods

Senescent cells have several morphological and biochemical characteristics that are used for their detection in vitro and/or in vivo [31,32]. Because no single marker is sufficient to unequivocally identify a senescent cell, combinations of markers and analytical techniques are typically used to increase the specificity of detection. Some markers that are frequently used for this purpose are listed below in Table 1. As mentioned previously, senescent cells are usually large and flat, and these morphological characteristics can be observed using light microscopy or flow cytometry (FC) [33,34]. Other techniques commonly used to detect senescent cells include immunofluorescence (IF), immunohistochemistry (IHC), Western blotting (WB), reporter assays, dye incorporation, enzymatic staining, PCR (polymerase chain reaction), FISH (fluorescence in situ hybridization), and ELISA (enzyme-linked immunosorbent assay). The most suitable detection method will depend on the objectives of the study and the chosen senescence model. Below we briefly describe some of the most important markers of each type.

### 2.1. Structural Change-Based Markers

The first major group of senescence markers consists of markers associated with structural changes in aging cells. In addition to the previously mentioned changes in cellular size and shape, senescence is often accompanied by an increase in lysosomal activity that can be detected by enzymatic staining [32]. A particularly notable senescence-associated lysosomal enzyme is senescence-associated β-galactosidase (SA-β-galactosidase), which has a pH optimum of pH 6.0 [35,36,37]. For a long time, SA-β-gal was the gold standard for senescent cell detection, but recent studies have raised concerns about its specificity. For example, SA-β-gal is active in neurons [38] and expressed in developing embryos [39], but neither of these observations is thought to be associated with senescence. Consequently, SA-β-gal activity cannot be considered specific enough to identify senescent cells by itself, although it remains widely used because its activity is closely related to aging and cellular proliferation status. Another frequently exploited hallmark of senescence is SA-α-fucosidase activity, which increases with lysosomal activation during aging. Importantly, its upregulation may be more specific than that of SA-β-gal [40]. Additionally useful as a marker of senescence is lipofuscin—a yellowish-brown residue consisting of incompletely degraded or metabolized lipids in lysosomes—that accumulates in aging tissues. Its presence can be detected using light microscopy, by exploiting its autofluorescence, or by Sudan Black B (SBB) staining [41,42]. A new biotin-conjugated SBB was developed for sensitive lipofuscin detection (GL13) that can be visualized using a specific anti-biotin antibody was also recently reported [43].

Another class of sensitive senescence indicators consists of DDR gene products, whose expression is usually visualized by immunofluorescence [32]. The DDR protein most commonly used for this purpose is γH2AX phosphorylated at Ser-139, which accumulates at double-stranded DNA break sites [44] and enables the detection of double-strand break (DSB) repair pathway proteins. The formation of a DSB leads to the binding of the MRN complex (Mre11/Rad50/NSB1), which plays a key role in detecting DSBs [45] and recruits and activates the ATM (ataxia-telangiectasia mutated) and ATR (ATM and Rad3-related) protein kinases, [46] which can also be used as markers of cellular aging. Other notable markers in this group include 53BP1, which co-localizes with γH2AX [47]; MDC1, which facilitates the recruitment of ATM kinase and, thus, promotes further H2AX phosphorylation [48]; Rad17, which reacts to local replication stress [49] and telomere dysfunction-induced foci (TIF) [50]. The downregulation of telomerase and telomere shortening, which can be measured by qPCR or FISH, are also useful markers of replicative cellular aging [51].

Premature senescence can be induced in human and murine cells by oxidative stress resulting from exposure to oxygen and certain hydroperoxides. These and other stressors provoke ROS (reactive oxygen species) production and subsequent DDR, leading to senescence induction [52]. Unsurprisingly, several authors have also linked senescence to mitochondrial dysfunction because mitochondria are major ROS producers. However, the induction of senescence by oxidative stress seems to involve complex and poorly understood processes. A recent study identified mitochondrial ROS as essential senescence inducers on the basis that artificial mitochondrial depletion in models of senescence caused cell cycle arrest without triggering key hallmarks of senescence: there was no increase in SA-β-galactosidase activity, many SASP factors were absent, and there was no upregulation of p16 and p21 [53]. ROS, and mitochondrial ROS in particular, are thus, potential biomarkers of oxidative stress-induced senescence that can be detected by fluorimetry or flow cytometry [54].

Epigenetic changes are also promising markers of senescence in human cells [55]. A notable example is the formation of senescence-associated heterochromatin foci (SAHF), which are heterochromatin domains that contribute to the silencing of proliferation-promoting genes in senescent cells [56]. SAHFs are easily detectable by DAPI or Hoechst 33342 staining and subsequent confocal microscopy visualization [32]. These thick structures bind to several proteins including heterochromatin protein 1 (HP1) and the methylated form of histone H3K9 [57], which can be detected by IF and could thus also serve as senescence markers. Another interesting senescence-associated epigenetic change is the formation of DNA-SCARS (DNA segments with chromatin alterations reinforcing senescence). These chromatin structures may be near-universal senescence markers because they appear in a majority of senescence subtypes and bind to several proteins related to DDR and promyelocytic leukemia protein (PML) bodies [58].

The structure of the nuclear lamina changes during senescence, which can affect both nuclear morphology and gene expression. Accordingly, the loss of lamin B1 was observed during DNA damage-, replicative-, and oncogene-induced senescence in human and mouse cell lines. In addition, lamin B1 mRNA and protein levels in murine tissues fell during irradiation-induced senescence experiments, making this protein another valuable senescence marker. The content of lamin B1 in the nuclear membrane can be determined by qPCR, IF, or WB [59].

#### Cell Cycle Arrest-Based Markers

There are some important negative markers associated with cell cycling that should be absent in senescent cells. In particular, assays based on the incorporation of bromodeoxyuridine (BrdU) [60] and/or 5-Ethynyl-2’-deoxyuridine (EdU) [61] should show low or absent DNA synthesis, and there should be no expression of Ki67, a protein that is exclusively expressed in proliferating cells and is present during all cell cycle phases except G0 [62].

As might be expected, proteins belonging to the p16/RB and p53/p21 senescence induction pathways are also common senescence markers. In particular, the overexpression of p16INK4a, pRB, phospho-pRB, or p21, p53, and phospho-p53 can be determined by WB, IHC, and/or IF [63]. Less well-known molecules from these pathways, such as DEC1 and PPP1A, have also been identified as potential biomarkers. DEC1 is a basic helix–loop–helix transcription factor that mediates p53-dependent premature senescence [64], while PPP1A is a catalytic subunit of PP1α that is active in the p53-mediated pathway during oncogene-induced senescence (OIS) [65].

### 2.2. SASP-Associated Markers

Some studies have tracked cytokine secretion associated with the SASP, which is characterized by the extensive secretion of pro-inflammatory compounds [66]. The secretion of SASP factors into the microenvironment of tissues is induced during damage- or oncogene-induced senescence and can be detected by WB, ELISA, or SASP-specific assays. The detection of several SASP-associated compounds and structures has been discussed in recent publications, but confirming senescence based on these markers alone is very challenging and the results obtained can be misleading because these substances are also secreted by non-senescent cells under some conditions. The SASP Atlas protein database was recently made available to address this issue [67]. Common SASP factors secreted by senescent cells include signaling molecules such as interleukins (e.g., IL-6 and IL-8), membrane-shaded adhesion molecules [68], and other growth factors [69].

### 2.3. Other Markers

A final important group of senescence markers consists of plasma membrane-associated proteins such as ICAM-1 or DEP1, which are expressed strongly during senescence [70]. In addition, senescent cells exhibit elevated levels of pro-survival/anti-apoptotic proteins such as Bcl-2 and Bcl-X. The suspension of apoptosis can be confirmed by detecting an absence of Annexin V or the cleaved forms of certain caspases [6,71].

It is important to note that there are ongoing efforts to identify alternative and more specific senescence markers, so a comprehensive list of all reported markers would be very difficult to compile. However, the preceding discussion and the table below cover all of the main biomarker groups and name several important biomarkers from each group.

### 2.4. Probes for Tracing Senescent Cells

Many fluorescent probes have recently been designed to monitor β-gal activity in senescent cells in vitro. Initially, they were validated only on human cells transfected with plasmids bearing the *Escherichia coli lacZ* gene. However, such models cannot reproduce the endogenous cellular β-gal activity caused by senescence. The first fluorescent probe for tracking senescent cells in vitro was tested in human diploid fibroblasts in replicative senescence. This probe emits blue-to-yellow light in response to β-gal activity and exhibits excellent photostability as well as low toxicity [72]. A few years later, Gal-Pro was introduced as a highly photostable fluorescent probe that responds rapidly and sensitively to β-gal activity in living cells undergoing oxidative stress-induced senescence [73]. These probes have different chemical structures but are similar in that they are initially non-fluorescent (OFF) in cells but are converted into fluorescent (ON) forms by the activity of β-gal, leading to the emission of detectable light. For in vivo imaging, one can use AHGa, which is an OFF/ON fluorescent probe containing a bond that is cleaved by β-gal in chemotherapy-induced senescent cells in mice [74]. Another recently developed probe for detecting senescent cells in cell culture and animal models is NIR-BG. This compound responded very well in drug-/radiation-/chemotherapy-induced senescent cells [75]. However, clinical applications of these probes are limited by their poor tissue penetration, which prevents detection by MRI or PET.

## 3. Cellular Models of Senescence

In vitro experiments are used to study biological phenomena in a controlled environment, potentially enabling the detailed analysis of multiple events simultaneously. However, cell and tissue culture flasks cannot perfectly replicate the external environments of cells in the tissues of an intact organism. There are two main ways of studying senescence in vitro: (1) by inducing senescence in the target cell line using exogenous factors, and (2) by using a cell line model designed for use in aging experiments or similar studies.

Replicative senescence in vitro is induced quite easily by simply passaging cells in culture. Due to constant telomere shortening [50,76], senescent cells are obtained in a few days or weeks, depending on the cell type and passage. Typical protocols for inducing replicative senescence in human diploid fibroblasts (e.g., WI-38 or IMR-90) include a culturing method, a splitting time, and confluence recommendations. However, all conditions and parameters must be optimized individually for specific cell types [32].

Multiple factors can initiate stress-induced or premature senescence [5]. The induction factor most commonly used in vitro and in vivo is radiation, which causes accelerated senescence in both cancer and normal non-malignant cells [77,78]. A key parameter in senescence induction by radiation is the dose: lower doses (0.5–10 Gy) cause senescence but higher doses (>10 Gy) can induce apoptosis. These responses depend on the severity of the radiation-induced DNA damage and DDR pathway activity [79]. Moreover, the effects of fractionated radiation can differ from that of the same quantity of radiation delivered in a single dose. For example, low-dose fractionated radiation-induced the p53/p21 pathway, resulting in a more significant senescence phenotype than a single higher dose even though the same total dose administered was identical [80]. Therefore, radiation-induced senescence depends on four crucial factors: (1) the dose rate, which affects the type and rate of DNA damage and the subsequent DDR; (2) the transformation and/or differentiation status of the targeted cells, e.g., cancer versus normal somatic cells may respond differently; (3) the cell type; and (4) the growth rate. Thus, the induction of senescence may differ among various cells in the same organ [81]. A typical protocol for inducing senescence with radiation involves applying a 10 Gy dose followed by light microscopy monitoring of morphological changes. Senescence markers are subsequently analyzed, but no sooner than from 7 to 10 days after irradiation [32].

ROS, such as hydrogen peroxide, superoxide anions, and hydroxyl radicals, are also important senescence induction factors because the oxidative stress resulting from ROS exposure causes damage [82] that can provoke senescence in human and rodent cells [52,83]. ROS production is triggered by exogenous and endogenous sources. The most important source is mitochondria, whose dysfunction has repeatedly been linked to aging and senescence [84]. The ROS generator most used to induce senescence in vitro is hydrogen peroxide. However, when using H_2_O_2_ for this purpose, its concentration must be optimized for each cell type individually by performing dose-response experiments to avoid cell death; high ROS concentrations promote apoptosis whereas lower doses activate the p53/p21 pathway [85]. Senescence is induced by incubating cells in a growth medium containing an appropriate H_2_O_2_ concentration (typically in the range from 50–800 μM) for four days. This treatment is then repeated and the shape of the cells is monitored; the onset of premature senescence is indicated by the detection of morphological changes resembling those associated with replicative senescence [86].

Senescence is one of several anti-neoplastic defense mechanisms in cells; instead of transformation, cells may enter a so-called intermediate state and become senescent. This process can be imitated in the laboratory, enabling the artificial induction of a senescence-like state through oncogene activation combined with the accumulation of tumor suppressors such as p53 and p16. The oncogene Ras can be used for this purpose [87]; a Ras-based protocol producing retrovirus-containing cells within six days has been developed involving transduction using the HRASv12 retroviral vector followed by puromycin selection and monitoring of typical senescence markers [88]. It was also reported that oncogene-induced senescence (OIS) can be triggered by using doxycycline-inducible expression of mutant RASV12 in a BJ-Ras system [89]. Unfortunately, retrovirus manipulation requires a biosafety level 2 (BSL-2) facility, making these protocols difficult to implement in some laboratories. It is also not always easy to obtain ready-to-use senescent cell lines, although an aging cell repository exists [90].

## 4. Animal In Vivo Senescence Models

In vivo studies can confirm in vitro findings and represent an important intermediate step between experiments on cell cultures and clinical trials. Animal models, particularly murine models, are usually used to follow up on and evaluate in vitro findings. In addition, the results of in vivo experiments can provide otherwise inaccessible insights into senescence induction mechanisms and facilitate the identification of possible therapeutic targets. Importantly, animal models are based on intact organisms and, thus, include all of the effects that individual tissues and molecular pathways have on each other. In general, the artificial induction factors or induction systems used in murine models of senescence and/or aging are very similar or identical to those used in cellular models. Because different murine models and methods of inducing senescence have different qualities and effects, care must be taken to ensure that the chosen model is well-suited to the planned experiments and their objectives.

Animal models of senescence often use naturally aged mice (mostly ≥ 2 years old) [8], but this approach is inevitably time-consuming. Consequently, artificial murine models exhibiting accelerated aging have been developed. These models have genetic characteristics similar to those seen in human progeroid syndromes, such as Hutchinson–Gilford progeria syndrome (HGPS), Werner syndrome, XFE progeria, or Trichothiodysthrophy [91], and undergo premature aging that resembles natural aging. These genetically modified animals exhibit the abnormal expression of genes encoding cell cycle checkpoint components (Cdkn2a, p53, Bub1b, etc.), proteins involved in DNA repair and maintenance (Ercc genes, Polg, Terc, etc.), proteins affecting nuclear mechanical properties (Lmna), ROS scavenging enzymes (Sod2), and signaling pathway components (Gsk3a) [92]. For many such models, senescent cells can only be detected during end-point analyses using euthanized animal tissues. Methods that have been used include qRT-PCR analysis of p16 or p21 in snap-frozen tissues, measurement of SA-β-galactosidase activity in fresh tissue samples, and ISH of p16 in fixed samples. Other markers such as ATM, ATR, H2AX, p53 or p21, and/or SASP factors are also sometimes examined by immunoblotting. Alternatively, murine blood can be collected to determine the expression of p16 in peripheral blood T lymphocytes, or plasma/serum can be sampled to evaluate senescence-associated changes in the secretion of SASP factors such as chemokines, cytokines, and matrix metalloproteinases using ELISA-based methods [93]. Long-term in vivo monitoring of senescence markers, such as p16, can also be achieved through imaging. The most famous model used for this purpose is known as the p16LUC mouse. These animals express the luciferase gene under the control of the p16INK4a promotor throughout their genome. Luciferase is, therefore, expressed whenever the p16 promotor is activated during senescence induction and can be detected by injecting the mice with the substrate luciferin, whose processing by luciferase generates measurable luminescence. In this case, euthanasia is performed only after the target marker is detected and the induction of senescence has been confirmed, unlike in other methods [94].

Premature aging can also be induced in mice by treatment with ionizing radiation. It is generally accepted that there is a close relationship between radiation sensitivity and aging. A dose from 5–10 Gy is typically used, depending on the irradiation susceptibility of the mouse strain in question. A single or a fractionated dose serves as the senescence induction factor. Depending on the aims of the experiment, one may irradiate the whole animal or just a part of the body, which is gentler in general. Two to six-month-old mice are suitable for irradiation, and non-irradiated mice can be used as controls. Alternatively, one can use two animal groups of different ages, e.g., 2-month-old animals as a young group and 24-month-old animals as old controls. The organs most frequently collected for examination are the lungs, liver, and kidneys, although the brain is sometimes a useful source of information. The isolation of nucleic acids followed by PCR analysis may be performed to obtain information on changes in gene expression. In addition, Western blot analysis may be used to clarify changes in protein levels, IF can be used to perform immunological analysis, and classical SA-βgal assays or measurements of BrdU incorporation may be helpful in some cases [95,96,97,98].

Recently, we have developed the method to obtain mouse and human hair follicular cells for genotyping, quantitative PCR, and quantitative immunofluorescence. The follicular cells were conveniently and non-invasively used for routine genotyping and the monitoring of cellular senescence in natural and experimentally (radiation)-induced aging [99].

Other ways of inducing artificial senescence are less commonly used in vivo. Oxidative stress is rarely used as an induction factor in mice, except for the D-galactose model. D-galactose serves as an artificial senescence inducer in animal models. Increased D-galactose induces ROS production and decreases antioxidant enzyme capacity. This particularly affects the brain, leading to memory loss, learning impairment, cognitive dysfunction, senescent cell accumulation in the brain tissue, shorter lifespan, and weakness, all resembling the aging phenotype [100,101,102]. The mechanism of D-galactose-induced aging is not clear, but recent studies indicate the accumulation of advanced glycation end products and ROS int tissues and cells, for instance, erythrocytes [103]. Usually, 2- to 3-month-old mice are administrated with a daily injection of D-galactose (100–500 mg/kg/day) subcutaneously for 7–12 weeks. The animal model is most frequently used for the functional testing of memory affected by senescent cell accumulation and their possible elimination with senolytics [104,105,106,107]. Importantly, the D-galactose senescence induction model was also validated in vitro [108], and relevant biomarkers such as p16, p21, p53, and/or SA-β-gal were detected [109]. Controlled oncogene activation to induce senescence in animals presents several difficulties; depending on the overall conditions, Ras-activating mutations may cause either proliferation or senescence in mice. This was demonstrated in a study using doxycycline-inducible transgenic mice, in which the outcome was shown to depend strongly on the Ras activation level, low activation promoted proliferation, while high activation triggered cellular senescence. These results indicate that Ras can be used to induce artificial senescence in vivo but its activation must be titrated very carefully [110]. In addition to the doxycycline-based transgenic model, Ras activation is used in a model designed for lung cancer research where the oncogene is activated by the intranasal administration of viral precipitates [111].

A decade ago, the CDK4/6 inhibitors (abemaciclib, palbociclib, ribociclib) were introduced to the clinic for the treatment of solid tumors. Those compounds induce G1 cell cycle arrest in cells and tissues [112,113,114,115]. The effect on treated cells depends on various factors, but in general, the compounds induce quiescence or senescence. Quiescent cells retain the capacity to exit the cell cycle arrest, whereas senescent cells perform a highly stable or permanent phenotype. CDK4/6 inhibitors were proven as quiescence and/or senescence inducers in vitro and in vivo in many studies [116,117,118,119,120,121,122,123,124,125]. A widely used protocol for senescence induction in mice consists of 14-week-old mice treated with abemaciclib administrated intraperitoneally [126] and palbociclib [116] or ribociclib applied by oral gavage [127]. Although cellular and animal models indicate the specific role of senescence in both the efficacy and toxicity of CDK4/6 inhibitors, the importance of senescence in clinical settings remains to be clarified.

## 5. Premature Aging Therapy

Aging is closely connected to an increased risk of many chronic diseases (including heart failure, myocardial infarction, dementia, strokes, many cancers, diabetes, blindness, or metabolic, renal, lung and bone dysfunctions), geriatric syndromes (frailty, sarcopenia, falls, incontinence, and cognitive impairment), the general deterioration of physical condition (leading to prolonged recovery from injury and increased incidence of infection), and mortality [128]. Because senescence contributes to many age-related pathologies [129], there is great interest in alleviating or eliminating the burden of senescence. This has led to the emergence in the last ten years of a new scientific discipline called senotherapy that focuses on aging, life extension, and quality of life while aging [130]. Key objectives in senotherapy are to increase the human lifespan and rejuvenate the function of certain tissues. The elimination of senescent cells from cell cultures, tissues, and even whole organisms may be possible with the assistance of senolytic drugs [131]. At present, major research areas within senotherapy include (i) searching for new senolytic drugs; (ii) determining the efficacy, selectivity, side effects, and other features of senolytic drugs in vitro, in vivo, and more recently, in humans; (iii) identifying effective drug delivery strategies; (iv) optimizing dosing and drug administration schedules; (v) identifying optimal markers of senescence to monitor therapeutic efficacy. Interest in this field has grown rapidly as potential clinical applications have emerged, and many start-ups and biotech companies have invested resources, time, and money into senolytic drug research [132]. Because this field is evolving rapidly, new findings are frequently reported, and new terminology has emerged. A glossary of commonly used terms relating to senescence is presented in Table 2.

### 5.1. Senolytic and/or Senotoxic Small Molecules

**Figure 4:**
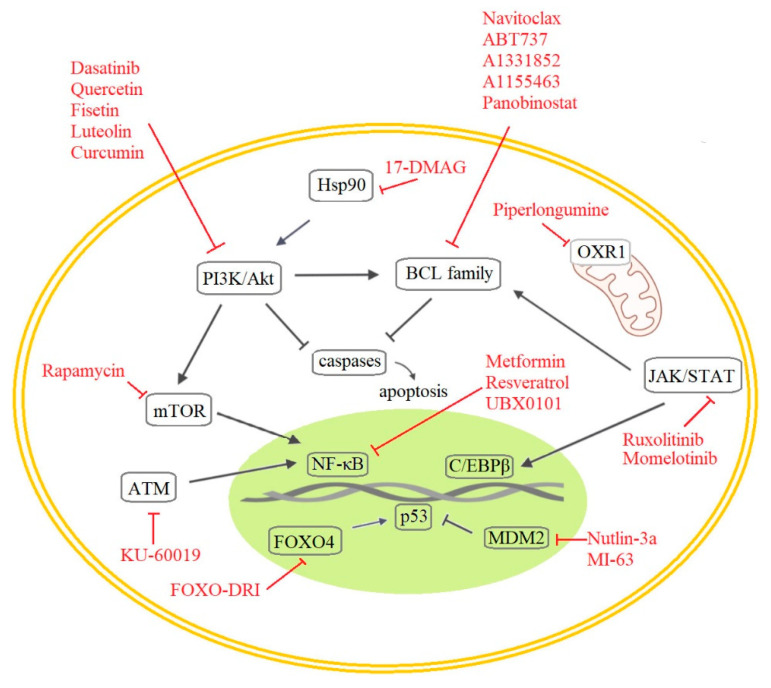
Molecular targets of senolytic drugs.

When considering senolytic drug research, a few major therapeutic approaches and targets stand out. Logically, the first approach proposed in this area involved targeting the senescent cells themselves, in particular their resistance to apoptosis [133] and other pathways involved in senescence. However, efforts have also been made to target the SASP, which is a key characteristic of senescent cells that is regulated independently from cell cycle arrest. [134] In addition, it is possible to induce the immune system to target senescent cells [135], and it may be possible to revoke the irreversibility of senescent cell fate [136]. Until recently, the cell cycle arrest of senescent cells was considered irreversible, but new studies have demonstrated they can re-enter the cell cycle in tumors [137] or can be reprogrammed into pluripotent stem cells [138]. As previously discussed, cells respond to oncogene activation with cellular senescence activation, but it is not always permanent. Cells may enter cell cycle again after some time spent in senescence, particularly if they gain epigenetic alterations derepressing the expression of anti-senescence genes, for instance, hTERT [139]. Tumor senescence is also one of the strategies of cancer cells to avoid the cytotoxic effects of anticancer therapy allowing survival in dormancy. Unfortunately, this may result in reservoir of tumor cells for future disease recurrence [140]. With regards to possible anticancer therapy, we can mention senescence key signaling molecules such as p16, p21, and p53 that also operate as regulators of stemness, because a gain of stemness in cancer cells can influence tumor aggressiveness and clinical outcome. It was shown that senescence-associated stemness is a detrimental feature helping cells to exit from cell cycle arrest and cause tumor recurrence [137,141]. Therefore, potential future use of senolytics includes anticancer therapies [142,143]. The most important targets of senolytic drugs identified to date are illustrated in Figure 4.

Senescent cells are characterized by the activation of anti-apoptotic pathways involving the BCL-2 protein family, p53, and the PI3K/AKT axis. Consequently, many current senolytic drug candidates target these proteins.

#### 5.1.1. Anti-Apoptotic Pathways

The inhibition of BCL-2 family proteins causes selective apoptosis in senescent cells. Navitoclax (ABT263) and its paralogue ABT-737 are first-generation senolytic drugs that inhibit BCL-2, BCL-X_L_, and BCL-w and thereby induce apoptosis in diverse cell types [6,144,145]. Both drugs also deplete senescent cells in vivo in sublethal irradiated and/or naturally aged mice [6,146], and Navitoclax suppresses SASP factor release in cells from old mice [146]. An unfortunate drawback of these compounds is their toxicity towards neutrophils and platelets, which limits their potential for clinical development [147]. This problem prompted the development of second-generation BCL-2 family inhibitors such as A1331852 and A1155463, which cause apoptosis in some human cell types [148]. Another notable anti-apoptotic target for senolytic drugs is the BH4 domain, which is the functional component of all anti-apoptotic protein family members [149]. Potentially useful starting points for developing new senolytic agents targeting this domain include the recently discovered alkaloid Piperlongumine (PL) [150] and its analogs Geldanamycin, Tanespimycin, and Alvespimycin [151]. PL is a natural compound isolated from trees of the *Piper* genus that preferentially kills oncogene activation-/radiation-/replicative stress-induced senescent cells. Its effects were initially attributed to the induction of apoptosis [152], but more recent results suggest that it targets OXR1 (oxidation resistance 1), a protein that is upregulated in some senescent human cells and which regulates the expression of many antioxidant enzymes. PL binds OXR1 directly, leading to its degradation [153]. A final notable anti-apoptotic senolytic drug is Panobinostat, which suppresses Bcl-X_L_ expression in chemotherapy-induced senescent cells [154].

#### 5.1.2. PI3K and Other Kinases

The first generation of hypothesis-driven senolytics includes some well-established small molecule drugs such as Dasatinib (D) and the flavonoid Quercetin (Q), which target multiple tyrosine kinases and PI3K, respectively. Dasatinib was originally an anti-cancer agent but also has senolytic effects in human senescent adipocyte progenitors. Quercetin is a natural compound with senolytic effects in umbilical vein endothelial cells. Their individual and combined senolytic effects have been demonstrated in vitro and in vivo [155]. Another compound in this group is the naturally occurring flavone Fisetin, which inhibits the PI3K/Akt pathway and has a stronger in vitro senolytic effect than quercetin, while also displaying lower toxicity and more selective Bcl-xL inhibition than Navitoclax. Other notable flavones are luteolin and curcumin, which performed well when tested together with Fisetin as a combined senolytic treatment [148,156]. However, the D + Q combination provided better overall senolytic performance.

#### 5.1.3. The p53 and p16 Axis and The DDR Pathway

As mentioned in the preceding sections, the p53 pathway plays a key role in regulating apoptosis and senescence and is, therefore, an attractive target for senotherapy. FOXO (Fork head box O) transcription factors are involved in diverse cellular functions including the induction of cell cycle arrest and senescence following interactions with p53 [157]. It was recently shown that the D-retro inverso (DRI)-isoform of FOXO4 can perturb the interaction of FOXO4 with p53 and thereby cause p53 to be excluded from the nucleus, triggering apoptosis in senescent cells both in vitro and in aged mice [150,158]. High-throughput screening of a small library of compounds in Ercc1^−/−^ mouse embryo fibroblasts (MEFs) with diminished repair capacity also revealed HSP90 inhibitors to be potentially useful senolytic agents: the HSP90 inhibitor 17-DMAG reduced p16 expression in both MEFs and a human progeroid syndrome model (Ercc1^−/Δ^ mice) [159]. Another screen identified the novel ATM inhibitor KU-60019 as a drug that alleviates senescence. ATM regulates lysosomal acidification, and treatment with KU-60019 reduced SA-β-galactosidase activity eliminated dysfunctional mitochondria and induced metabolic reprogramming in human fibroblasts. In addition, treatment with this compound facilitated wound healing in aged mice [160].

Another approach to senolytic discovery involves targeting a different characteristic of senescent cells—the SASP, i.e., their secretion of growth factors, chemokines, and cytokines. Under certain conditions, suppressing the deleterious paracrine and autocrine SASP effect can be more beneficial than affecting cell cycle arrest [134]. Many signaling pathways are at least partially involved in SASP regulation (mTOR, MAPK, PI3K, and GATA4/p62). They intersect in the induction of NF-κB and the CCAAT/enhancer binding protein beta (C/EBPβ) pathway, which offers a wide range of targets for senotherapy [66]. NF-κB is the major signaling pathway involved in SASP activation [161].

#### 5.1.4. NF-κB and C/EBPβ Regulation

Senolytic drugs can also directly target NF-κB or C/EBPβ, or upstream regulators of NF-κB. For example, the serine/threonine kinase mTOR influences IL-1α expression and activates NF-κB via its interactions with the MAPK pathway. Accordingly, the mTOR inhibitor rapamycin was recently shown to cause the partial suppression or degradation of SASP components [162,163]. MAPK inhibitors (primarily p38MAPK inhibitors) have also been tested for senolytic activity but have only shown interesting results in some cell types or induced at best partial senolytic effects [163,164]. However, the activity of NF-κB can be suppressed by Nutlin-3a in a p53-dependent manner because it inhibits Mdm2. Similar results were obtained with two other Mdm2 inhibitors, UBX0101 and MI-63 [124,165,166]. In addition, metformin and resveratrol reduce the nuclear translocation of NF-κB, leading to the inhibition of some SASP components and have, therefore, been studied as potential senolytic drugs [167,168]. Another transcription factor influencing the expression of SASP components is C/EBPβ, which is regulated by JAK/STAT pathway activation [169]. The drugs Ruxolitinib (INCB18424) and Momelotinib (CYT387) target JAK/STAT and, thus, significantly reduced SASP secretion in radiation-induced senescent human preadipocytes, and Ruxolitinib also had an effect in 2-year-old mice [170]. Another option is to target a specific SASP component (e.g., a certain interleukin or its receptor) using a target-directed monoclonal antibody. Such antibodies have already been developed [171].

### 5.2. Other Approaches to Eliminate Aging Cells

We have recently performed a high-throughput automatized screening HTS of the commercial LOPAC^®^Pfizer library on aphidicolin-induced senescent human fibroblasts, to identify novel senolytics. We discovered the nociceptin receptor FQ opioid receptor (NOP) selective ligand 1-[1-(1-methylcyclooctyl)-4-piperidinyl]-2-[(3R)-3-piperidinyl]-1H-benzimidazole (MCOPPB, a compound previously studied as a potential anxiolytic) as the best scoring hit. The ability of MCOPPB to eliminate senescent cells in in vitro models was further tested in mice and in *C. elegans*. MCOPPB reduced the senescence cell burden in peripheral tissues but not in the central nervous system. Mechanistically, MCOPPB treatment activated transcriptional networks involved in the immune responses to external stressors, implicating Toll-like receptors [172].

Since oxidative stress was linked to many age-related pathologies and senescence itself, some studies researched the possible therapeutic impact of antioxidants. For example, vitamins A, C, and E [173,174] were intensively investigated for their possible antiaging effects. Unfortunately, those trials did not show any stable and convincing results supporting preventive vitamin use to reduce mortality [175]. The problem might be in targeting antioxidants to a particular tissue and also in antioxidant suitability for the damaging species. Another antioxidant compound, coenzyme Q10, plays an important role in mitochondrial respiration [176] and its deficit was associated with many pathological processes. Coenzyme Q10 is safe, well-absorbed, and distributed agent. Thus, it is frequently used in antioxidant strategies with reported symptomatic benefits [177]. Selenoproteins were also identified as antioxidant enzymes targetable with nutrients [178]. Several clinical trials indicated that selenium supplementation can prevent multiple diseases. However, there is also evidence of a higher risk of neuronal diseases after selenium administration as a consequence of its neurotoxic effects [179]. Therefore, it is necessary to balance selenium intake to maximize health benefits and minimize possible toxic effects [180]. Senolytics with antioxidant properties are also plant-derived polyphenols (quercetin and resveratrol) [181]. In addition to antioxidant agents, physical training balances oxidative stress and mobilizes antioxidative enzymes. Regular exercise of moderate intensity is one of the most important preconditions of health maintenance, reducing oxidative stress and avoiding chronic diseases progression [182,183].

The immune system also plays a vital role in eliminating senescent cells. However, aging impairs its functionality, rendering the clearance of senescent cells inaccurate. Senescent cells and/or immune cells releasing SASP factors with impact in cancer progression are CD4 + T cells, CD8 + T cells, B cells, NK cells, and macrophages [184]. The elimination of senescent cells is mediated by CD4 + T cells and NK cells in a process known as senescence surveillance [135,185]. During this process, the NK cell receptor NKG2D binds ligands found on the surfaces of senescent, damaged, and stressed cells [186]. Consequently, NKG2D and other immune system components have become important targets in senotherapy.

Generally said, senescent cells attract the innate immune system by inflammatory factors’ secretion (components of SASP), which triggers the activation or suppression of a certain type of macrophages. Further, the inflammatory signaling recruits NK cells to senescent cells; NK cells have receptors binding ligands from senescent cells and this process ends with senescent cell death [187]. Interactions between senescent cells and macrophages were observed with different results and explanations over the past few years. Their exact interaction depends on precise SASP excretion, ligands presented on senescent cells, cell type, and the senescence inducing factor [67]. SASP components attract also NK cells leading to senescent cell clearance, which was proven in the murine model [188]. Current possible therapy for senescent cell clearance by the immune system is based on a few cell therapies for cancer, which might be considered as senolytic therapy [189]. Regarding senescent cancer cells, in vivo studies showed senescence induction in tumor cells. Senescent cell occurrence hampered tumor growth and also activated the immune system by SASP excretion, which can lead to the clearance of both senescent tumor cells and adjacent neoplastic cells with a beneficial impact on tumor regression [190,191,192]. Additionally, it is very important to emphasize the dual function of the immune system in cancer. Some chemokines can also attract inflammatory cells which promote tumor proliferation, angiogenesis, and invasiveness. Thus, opinions about the role of senescence in tumors differ and depend on context and surrounding conditions [193].

As the preceding discussion shows, many promising senolytic compounds (Table 3) have been discovered. However, an important question to consider is whether it is desirable to bypass or even revert cellular senescence. We know that it can be achieved; proliferation of senescent cells can be reactivated by enforced telomerase activity [136]. Moreover, OIS can be bypassed by depleting specific interleukins or inhibiting specific chemokine receptors [194,195]. However, reversing senescence may be dangerous; a recent study found that senescent cells that re-enter the proliferation phase frequently develop into lymphoma or leukemia cells with enhanced stemness, potentially giving rise to highly aggressive tumors [141].

Because senotherapy is a rather new therapeutic field, new strategies will be needed to translate discoveries resulting from laboratory experiments into drug candidates before clinical trials can begin. However, the discovery of new senolytic compounds in vitro or in vivo is challenging.

### 5.3. Senolytics in Anti-Cancer Therapy

Growing evidence supports the use of senolytics in complex anti-cancer therapy. Particularly interesting would be a combination of therapy-induced senescence and the subsequent elimination of remaining and therapy-resistant aged cells by senolytic drugs [142,196,197]. Proof-of-concept data were already shown for ABT263 (navitoclax) treatment of chemotherapy-induced senescent cells [198]. ABT263 was successfully tested in etoposide or doxorubicin-induced in vitro and animal models [155,198,199,200] and also in radiation-induced breast and lung cancer cellular models [200]. The application of ABT263 in animal models decreased the development of metastasis and occurrence of relapse [201]. The structurally similar compound, ABT737, eliminated senescent cancer cells in a radiation-induced model of cell lines and xenografts [202] and also showed efficacy in etoposide-based chemotherapy in breast cancer cell lines [203]. Another BCL family inhibitor, Nav-Gal (galacto-conjugated navitoclax), was effective in senescence models induced by several anticancer agents: palbociclib, doxorubicin and radiation, and/or with cisplatin [204]. In addition, digoxin and ouabain (Na^+^/K^+^ pump inhibitors) affected many types of cancer cell lines and xenografts after senescence induction [158,205]. Another class of anticancer and senolytic agents used clinically are mTOR inhibitors, which showed efficacy in drug-induced senescent cancer cell lines and xenografts [206,207]. Last but not least, there are attempts to employ immunological strategies, for instance, blocking antibodies and CAR T cells to target senescence in tumor cells and animal models [117,208,209,210,211]. In summary, the abovementioned studies indicate that a two-step approach of pro-senescence anticancer therapy followed by senolytic treatment is a feasible strategy in curing human cancers [198]. However, we need to have more clinical trial data to implement those strategies into routine practice.

### 5.4. Nanoparticles for Delivery of Active Compounds into Senescent Cells

Nanoparticles (NPs) offer a new delivery strategy for targeting senescent cells in vitro and in vivo with potential diagnostic and therapeutic applications. Importantly, NPs can be used to encapsulate small molecules such as senolytic drugs, while also being coated with substances that facilitate their delivery to specific tissues or cell types of interest. Some very effective NP-based technologies for packaging and transporting loads to targets are available. However, problems may arise in vivo relating to the interactions, distribution, side effects, and clearance of the NPs. These tools, thus, remain challenging to use well and require further study.

The first reported strategy for targeting senescent cells with NPs relied on 100 nm spherical beads containing rhodamine on an MCM-41 silica matrix that was coated with galactooligosaccharides (Gos) of various lengths. The beads were taken up into cells by endocytosis and released via exocytosis, and the GosNPs preferentially fused with lysosomal vesicles in senescent cells. The coating was subsequently improved by using homogenous 6-mer galacto-oligosaccharides (Gal) and the resulting NPs were validated in damage-/chemotherapy-induced senescence models [212,213]. CaCO_3_ NPs carrying rapamycin also showed senolytic activity. These NPs were coated with a conjugate of lactose with polyethylene glycol and an anti-CD9 monoclonal antibody (the CD9 receptor is strongly expressed in senescent cells) to improve their targeting [214]. In addition, molybdenum disulfide NPs (MoS_2_ NPs) inhibited H_2_O_2_-induced senescence by halting lysosomal and mitochondrial dysfunction [215].

## 6. Clinical Trials (CTs)

Based on promising preclinical results in murine models, a few senolytics (identified from a group of 46 compounds showing potential senolytic effects) have been included in clinical trials in humans for the treatment of age-related pathologies. As the result of promising preclinical data (on mouse models), currently, some senolytics (from 46 compounds identified as having a possible senolytic effect) undergo clinical trials in humans for age-related pathology treatment. Unsurprisingly, the compounds with the greatest potential appear to be natural products and/or previously approved drugs. The outstanding and most heavily studied agents in this group are dasatinib and quercetin (individually or in combination) along with metformin, fisetin, and UBX0101. Early preclinical studies using aging and disease models have provided very promising results [216,217]. However, further work will be needed to clarify the potential of these prospective senolytics.

## 7. Conclusions

Because of the challenges posed by aging populations, there is a pressing need for drugs that can enable people to remain healthy as they age and to enjoy a long lifespan with a good quality of life. Consequently, there is great interest in cellular senescence and the development of senolytic drugs that can eliminate senescent cells or reverse senescence. This review summarizes current knowledge about senescence biomarkers, cellular and animal models of senescence, and the early development of senolytics. Challenges remaining to be overcome and areas where further clarity is needed have been highlighted; overcoming these challenges will enable the development and clinical testing of more effective senolytic medicines. The main barriers that must be overcome to enable the clinical use of senolytic drugs include our currently limited understanding of their targets and pathways, a limited range of animal models, a lack of highly selective therapies and probes, and the limited availability of data on the biodistribution and elimination of both senolytic drugs and nanoparticle vehicles for their delivery. Despite these challenges, the field of senescence research is vibrant and has the potential to ultimately offer personalized solutions for increasing human longevity.

## Figures and Tables

**Table 1:** Notable senescence markers and methods used for their detection.

Senescent Cell Feature	Biomarker	Marker Level Change	Senescence Type	Detection Method
morphological changes	morphology, size	wide and flattened	general	light microscopy, FC
lysosomal activity	SA-β-galactosidase	increased	general	enzymatic staining
	SA-α-fucosidase	increased	general	enzymatic staining
	Lipofuscin	increased	general	SBB, GL13
DNA damage, DDR	γH2AX	increased	general/damage-induced	IF
	Mre11	increased	general/damage-induced	IF
	Rad50	increased	general/damage-induced	IF
	NSB1	increased	general/damage-induced	IF
	ATM	increased	general/damage-induced	IF
	ATR	increased	general/damage-induced	IF
	53BP1	increased	general/damage-induced	IF
	MDC1	increased	general/damage-induced	IF
	Rad17	increased	general/damage-induced	IF
	TIF	increased	general/damage-induced	IF
low/lack of DNA synthesis	BrdU	decreased	general	staining incorporation, IF
	EdU	decreased	general	staining incorporation, IF
lack of proliferation	Ki67	decreased	general	IHC, IF
p16/pRB pathway	p16INK4a	increased	general	WB, IHC, IF
	pRB	increased	general	WB, IHC, IF
	phospho-pRB	increased	general	WB, IHC, IF
p53/p21 pathway	p53	increased	general/damage-induced	WB, IHC, IF
	p21	increased	general/damage-induced	WB, IHC, IF
	phospho-p21	increased	general/damage-induced	WB, IHC, IF
	DEC1	increased	general/damage-induced	IHC, IF, special assay
	PPP1A	increased	general/damage-induced	IHC, special assay
ROS	ROS	increased	general/oxidative stress-induced	fluorometry, FC
telomere length	telomere	decreased	replicative-induced	qPCR, FISH
SAHFs	SAHFs	increased	general/damage-induced	DAPI/Hoechst, confocal microscopy
	HP1-gamma	increased	general/damage-induced	IF, IHC
	H3K9-methylation	increased	general/damage-induced	IF
	PML bodies	increased	general/damage-induced	IF
nuclear membrane	lamin B1	decreased	general	WB, IF, qPCR
cytokine secretion	SASPs	increased	damage-/oncogene-induced	WB, ELISA, SASP-assay
others	plasma membrane proteins	increased	general/replicative-/oncogene-induced	IF, WB, IHC, FC
	apoptosis elimination	absent	general	IF, IHC

**Table 2:** Basic glossary of new terms associated with senescence and senotherapy.

Term	Description
Senescence	Biological aging. Process of senescent cells (SCs) accumulation, SCs do not function, but they are metabolically active and remain in tissues. SCs are closely associated with age-related disorders.
Senotherapy (Senolysis)	Removal of senescent cells.
Senolytic drugs (Senolytics)	Class of drugs selectively eliminating SCs.
Senoblockers	Agents affecting epigenetic regulators to reactivate programs of youthfulness and regeneration.
Senomorphics	Small molecules inhibiting SASP.
Senostatics	Drugs interfering cells entering to senescence.
Senomodulators	Drugs suppressing SASP activity.
Senosuppressors	Therapeutics slowing down SCs accumulation rate.

**Table 3:** List of senolytic drugs.

Senolytic Drug Targets	Compound	Target	Note
Anti-apoptotic pathway	Navitoclax	BCL-2, BCL-X_L_, and BCL-w	ABT263
	ABT-737	BCL-2, BCL-X_L_, and BCL-w	ABT263 paralogue and precursor
	A1331852	BCL-X_L_	2nd generation of BCL-2 family inhibitors
	A1155463	BCL-X_L_	2nd generation of BCL-2 family inhibitors
	Piperlongumine	apoptosis	An alkaloid, dietary natural product from *Piper* genus trees
	Geldanamycin	?	Piperlongumine analogue
	Tanespimycin	?	Piperlongumine analogue
	Alvespimycin	?	Piperlongumine analogue
	Panobinostat	BCL-X_L_	increases 3/7 caspase activity
PI3K and other kinases	Dasatinib	PI3K/Akt pathway	small molecule inhibiting various tyrosine kinases
	Quercetin	PI3K/Akt and mTOR pathway	Flavonoid
	Fisetin	PI3K/Akt	natural flavonoid
	Luteolin	PI3K/Akt	Flavone
	Curcumin	PI3K/Akt	Flavone
p53, p16, and DDR pathway	FOXO4-DRI	FOXO4 and p53 interaction	mitochondrial activity boost
	17-DMAG	HSP90/Akt	SASP suppressor
	KU-60019	ATM	NF-κB inhibition
NF-κB or C/EBPβ regulation	Rapamycin	mTORC1 complex	next generation Mdm2 inhibitor
	Nutlin-3a	Mdm2	p53 stabilization and SASP reduction
	MI-63	Mdm2	p53 stabilization and SASP reduction
	UBX0101	Mdm2	derived from Nutlin
	Metformin	SASP	NF-κB inhibition
	Resveratrol	SASP	NF-κB inhibition
	Ruxolitinib	JAK	INCB18424, C/EBPβ repression
	Momelotinib	JAK	CYT387, C/EBPβ repression
Other	MCOPPB	NOP	anxiolytic opioid

## Data Availability

Not applicable.

## Search Strategy and Selection Criteria

The search strategy used when conducting this review was designed to identify and evaluate well-documented studies on various aspects of senescence including the identification of senescent cells in vitro and in vivo, potential senescence markers, and newly discovered senolytics. The PubMed, Web of Science, and www.clinicaltrials.gov databases (access date 1 March 2022) were searched, focusing on articles and entries published between 1961 and 2021. All searches were up to date as of January 2022. The search terms used included “senescence”, “senescence marker”, “senescence model”, “aging”, “senolytics”. Only contributions in English were considered. All figures were created using the Biorender application.
